# Special Issue “New Therapies of Liver Diseases”

**DOI:** 10.3390/jcm11071798

**Published:** 2022-03-24

**Authors:** Pierluigi Toniutto

**Affiliations:** Hepatology and Liver Transplantation Unit, Academic Hospital, University of Udine, 33100 Udine, Italy; pierluigi.toniutto@uniud.it

Medical and surgical treatments aimed at curing severe liver diseases and prolonging the survival of patients have improved dramatically in recent years. These advances have mainly been achieved by obtaining a better understanding of the pathophysiology of liver diseases [1]. New and old pharmacological therapies have been applied in a better way based on the new insights obtained in the pathophysiological studies. Moreover, the increased application of technology innovations, both in diagnostic imaging [2] and in surgery [3], enable the cure rate to be increased in patients with advanced liver diseases.

A Special Issue in the *Journal of Clinical Medicine (JCM)* has been dedicated to collecting high-quality scientific contributions from leading experts by focusing on updating the horizon of new pharmacological therapies and new surgical approaches that can be applied to cure several types of liver diseases as a method to address this challenging topic in greater detail.

Two studies investigated the current and future management of cholestatic liver diseases [4,5]. The first study retrospectively evaluated the real-world clinical management of patients with primary biliary cholangitis (PBC), according to the indications of the recent updated clinical guidelines. A study conducted in a large cohort of European patients revealed that biochemical response rates adopting the standard first-line treatment with ursodeoxycholic acid (UDCA) were achieved in a large proportion of patients, depending on the response criteria adopted. In UDCA nonresponders, second-line treatment regimens in which obeticholic acid or bezafibrate were added were promptly applied, leading to significantly increased response rates. These results confirm in real clinical practice that UDCA first-line standard treatment is largely effective in patients with PBC, but highlight the need to detect high-risk patients with an insufficient response to UDCA early in life, since early treatment modification significantly increases subsequent response rates. In addition to obeticholic acid and fibrates, several other molecules are currently under evaluation as potential new therapies both for patients with PBC and with primary sclerosing cholangitis (PSC). This issue has been extensively discussed in a subsequent study [5]. Given the complex nature of PBC and PSC, future treatments for these diseases will probably be based on a combination of drugs, aimed at influencing specific pathophysiological mechanisms in different stages of disease severity. 

The current therapeutic strategies for the management of patients with cirrhosis are focused on the prevention or treatment of specific clinical complications such as ascites, gastrointestinal bleeding and hepatic encephalopathy [6]. “Etiologic therapy”, which is designed to remove the causative agents of the disease (i.e., viruses or alcohol), prevents clinical decompensation in most patients with cirrhosis. In contrast, a significant proportion of patients with decompensated cirrhosis remain at risk of further disease progression despite the application of etiologic treatments. Thus, the identification of new therapies targeting specific key points in the complex pathophysiological cascade of decompensated cirrhosis is urgently needed. These therapies are presented in updated detail in this Special Issue of *JCM* [7]. Poorly absorbable oral antibiotics, statins, and albumin have been proposed as potential disease-modifying agents for cirrhosis (DMAC), since clinical studies have shown their capacity to prolong survival. The ideal DMACs candidate should be directed to modify the key mechanisms in the pathogenetic network of the gut-liver axis, such systemic inflammation, and immune dysfunction. 

The development of ascites is one of the typical complications of advanced cirrhosis. Treatment of non-tense ascites comprises the assumption of a combination of furosemide and anti-aldosterone diuretics, accompanied by a restrictive sodium and water diet [6]. Tolvaptan, a selective vasopressin type 2 receptor antagonist, was approved in some countries for treating ascites in patients who responded insufficiently to conventional diuretics. Several still unresolved questions persist regarding both the long-term efficacy of tolvaptan and its effect on the survival of patients with cirrhotic ascites. A recent Japanese retrospective study presented in this issue of *JCM* seems to show that the addition of tolvaptan prolongs the survival of patients with cirrhotic ascites compared to standard diuretic drug combination alone, especially when tolvaptan is started before high-dose furosemide administration [8]. Although these results are encouraging, further prospective studies in different countries must be performed to standardize the use of aquaretic drugs in treating cirrhotic ascites.

More challenging is the treatment of ascites when it reaches the stage of refractoriness. An updated and exhaustive analysis of this topic has been reported in this issue of *JCM*, including the more recent data regarding the placement of a transjugular intrahepatic portosystemic shunt (TIPS) and chronic albumin administration [9]. TIPS reduces portal hypertension, allows greater control of ascites, and in some cases improves the clinical course of the disease. Some concerns persist regarding both the correct selection of patients with ascites who may truly benefit from TIPS and the prevention of cardiac and neurologic complications after TIPS placement in the long term. The effect of long-term human albumin administration in treating grade 2–3 ascites has been studied in the ANSWER [10] and MATCH [11] randomized clinical trials, producing contradictory results. The different results might be at least partially explained by differences in disease severity of the patients enrolled (slightly less severe in the ANSWER trial) and dosage and duration of albumin treatment (higher and longer, respectively, in the ANSWER trial). Regardless, the long-term albumin administration in patients with persistent ascites remains not systematically used in various parts of the world, despite proved effectiveness both in ascites control and long-term survival. The placement of implantable ascites drainage devices has been experimented with contradictory results. To date, there is no clear indication to use these devices except as part of controlled clinical trials. 

In addition to ascites, gastrointestinal bleeding and the development of hepatic encephalopathy (HE) represent the other key determinants of the transition from clinically compensated to decompensated liver cirrhosis. Two contributions to the present issue of *JCM* are devoted to exploring these clinical complications of cirrhosis. The most recent advances in the management of esophageal variceal hemorrhage in cirrhotic patients have been updated [12]. These guidelines were derived from the applications of specific treatment algorithms involving the use of indirect measurement of portal pressure (HVPG) and the rescue placement of TIPS, in addition to vasoconstrictors, endoscopic band ligation of esophageal varices and antibiotic prophylaxis. Patients who may benefit more from early rescue TIPS placement are active bleeders with poor predictors of the response to standard medical treatment (Child C class, portal vein thrombosis, HVPG > 20 mmHg, and systolic blood pressure < 100 mmHg at admission). Although the use of preemptive TIPS in these patients has been recommended since the Baveno V consensus [13], only a minority of potential candidates finally undergo a preemptive TIPS. This finding indicates that preemptive TIPS is largely underutilized in real-life practice. This is a topic where it is probably necessary to implement scientific information in the community of hepatologists, in order not to lose the clinical and survival advantages that the correct indication of the positioning of the preemptive TIPS can bring to patients. 

HE in cirrhosis has profound implications in terms of the patients’ ability to fulfil their family and social roles, to drive and to provide for themselves. The past few years have been characterized by significantly more attention to HE and its implications. Its definition has been refined, and a small number of new drugs or alternative management strategies have become available, while others are underway [14]. Currently, overt HE is generally managed by the correction of any identified precipitating factors and institution of ammonia-lowering treatment with nonabsorbable disaccharides and nonabsorbable antibiotics [15]. Many therapies other than nonabsorbable disaccharides and nonabsorbable antibiotics have been studied. Among them, L-Ornithine L-Aspartate (LOLA), which is a substrate for the urea cycle and increases urea production in peri-portal hepatocytes, has been extensively studied. Despite promising results, a recent review and meta-analysis suggests that the effect of LOLA is comparable to other ammonia-lowering agents in treating HE regardless of clinical severity. The use of nonurea nitrogen scavengers (sodium benzoate, sodium, glycerol phenylbutyrate, and ornithine phenylacetate) has not been shown to be superior to placebo or to standard treatment in clinically improving HE. Muscle loss impacts nitrogen and ammonia metabolism and is associated with an increased risk of HE. Thus, the maintenance of adequate daily energy (35–40 kcal/kg ideal body weight) and protein intake (1.2–1.5 g/kg ideal body weight) has been associated with the improvement of psychometric performance and quality of life and with the reduction in the risk of overt HE development. In addition to small meals consumed throughout the day, a late evening snack comprising complex carbohydrates should be strongly recommended, as they reduce protein catabolism and interrupt the long fast between dinner and breakfast. The postulated efficacy of branched chain amino acids (BCAAs) administration in treating HE is probably a surrogate for an increase in the intake of proteins containing BCAAs, particularly in patients consuming vegetable diets.

If the development of ascites, gastrointestinal bleeding, HE or any combination of these conditions are the distinct features of acute decompensation of liver cirrhosis, acute-on-chronic liver failure (ACLF) is a distinct syndrome that develops in patients with acutely decompensated chronic liver disease and is characterized by a high 28-day mortality rate. Thus, a special article dedicated to ACLF has been presented in this issue of *JCM* [16]. The key elements identifying the appearance of ACLF are the strong association with precipitating factor(s), the development of single- or multiple organ failures (OFs) and an intense systemic inflammation. Excessive inflammation is responsible for tissue damage and for necrotic cell death, leading to the release of damage-associated molecular patterns (DAMPs) that maintain inflammation by binding to pattern-recognition receptors (PRRs). Although many of the pathophysiological mechanisms responsible for the development of ACLF have been elucidated, additional knowledge is needed to develop treatments besides supportive measures for OFs. To date, early liver transplantation (LT) produces good outcomes in a subset of patients presenting grade 3 ACLF, thus these patients must be early referred to a liver transplant center to verify the feasibility of liver transplant. 

Due to the availability of direct antiviral agents (DAAs) for curing hepatitis C virus infection [17], the only two major hepatitis viruses are still awaiting a definitive cure are hepatitis B (HBV) and hepatitis D (HDV). More recent advances in treating these viruses have been highlighted in another article presented in this issue of *JCM* [18]. The main endpoint of all current treatment strategies for these chronic infections is the suppression of HBV DNA and HDV RNA for those patients coinfected with HDV. Unfortunately, the profound suppression of viral replication does not translate to an effective and complete cure of HBV or HBV/HDV coinfection. Among the known barriers to achieve a “functional cure”, the most worrisome is HBV covalently closed circular DNA (cccDNA), which allows the virus to permanently persist in hepatocytes and against which nucleot(s)ide analogs have little effect. Regarding HDV infection, the ideal goal of treatment is to obtain simultaneously the clearance of HBsAg and a sustained HDV virological suppression, at least 6 months after stopping the treatment. Unfortunately, both aims are still not reachable. Improving knowledge of the structure and replication cycle of both HBV and HDV has facilitated the development of novel antivirals directly targeting multiple steps in virus replication and preventing the synthesis of new cccDNA. Furthermore, immunomodulators may also be needed to reverse the state of tolerance typical of the chronic phase of viral infection and subsequently promote the immune-induced death of infected hepatocytes, which is crucial for the neutralization of circulating virions. New nucleotide analogs in advanced phase of development are besifovir, metacavir and two prodrugs of tenofovir (tenofovir exalidex and tenofovir disoproxil orotate). Other drugs in development are the attachment/entry inhibitors, such as bulevirtide, which acts upon the sodium taurocholate co-transporting polypeptide (NTCP), a receptor of both HBV and HDV. Therefore, this new drug blocks both HBV and HDV entry in the hepatocytes. Bulevirtide was approved in the European Union in July 2020 as the first effective drug for the treatment of chronic HDV in patients with compensated liver disease. In addition to bulevirtide, a further new drug is lonafarnib (LNF), a farnesyl transferase inhibitor that blocks the assembly and secretion of virions in the cell through HDV antigen prenylation. Preliminary data seem to support the combined use of LNF with ritonavir (RTV). Nucleic acid polymers (NAPs), such REP 2139, are under clinical evaluation and produced promising results, as among 12 enrolled patients 7 have become HDV RNA- and 5 hepatitis B surface antigen (HBsAg)-negative, respectively, after a follow-up of 1 year.

Three contributions presented in this issue of *JCM* have been devoted to providing the main updated knowledge regarding the approach to treat patients with hepatocellular carcinoma (HCC) and cholangiocarcinoma (CCA). One article focused on CCA [19], and two focused on HCC [20,21]. CCA is anatomically classified in intrahepatic (iCCA), perihilar (pCCA) and distal (dCCA) CCA. Surgical resection, obtaining negative margins, represents the best curative therapy for CCA. Systemic treatment with cisplatin plus gemcitabine (GEMCIS) is the first-line approach for patients with advanced-stage CCA, but the results are unsatisfactory, with a 5-year survival rate of approximately 5–15%. Targeted therapies, specific molecular profiling and biomarkers are needed to select new effective therapies for each patient with CCA. For example, approximately 15–20% of iCCAs have been observed to contain FGRF2 translocations, which are implicated in promoting cell proliferation and angiogenesis. Thus, several FGFR 1–3 inhibitors (i.e., pemigatinib and infigratinib) are being evaluated in phase III trials involving patients with advanced CCA, and the preliminary results seem to be encouraging. Mutations in epidermal growth factor receptor (EGFR) have a great importance in guiding treatments in different cancers, nevertheless, no evidence of their efficacy against CCA has been demonstrated. Immune checkpoint inhibitors (ICIs), peptide- and dendritic cell-based vaccines, and adoptive cell therapy, are under investigation to treat patients with CCA. Although the use of immunotherapy in patients with CCA is still limited, several clinical trials are currently evaluating the therapeutic properties of anti-CTLA-4 monoclonal antibodies, the targeting of PD-L1 or its receptor, PD-1, as well as chimeric antigen receptor T (CAR-T) cell immunotherapy. Unfortunately, ICI monotherapy has shown insufficient efficacy in patients with CCA. However, a better understanding of immunologically based therapeutic strategies should be reached, before to design a real precision medicine strategy allowing to reduce clinical aggressiveness of the tumor and to improve the prognosis of patients with CCA.

Compared to patients with CCA, very different treatment scenarios are on the horizon for patients with HCC. In addition to the well-known therapeutic options referring to surgical resection or to locoregional treatments of the tumor, a very large quantity of data is expected from new systemic treatments based on the use of ICIs in combination with other agents, among which vascular endothelial growth factor (VEGFR)-targeted therapies generated very encouraging results. Therefore, atezolizumab (a monoclonal antibody against PD-L1) plus bevacizumab (a monoclonal antibody against VEGF) has been approved as the first-line treatment option for advanced HCC, becoming the standard of care for these patients. Immunotherapy-based treatments will increase the landscape of HCC therapy soon. A very attractive first-line treatment modality in patients with intermediate-stage HCC is to combine locoregional treatments with ICIs, since ablative and intraarterial techniques indirectly induce a peripheral immune response that may enhance the effect of ICIs. Both radiofrequency ablation and transarterial chemoembolization induce necrosis of tumor cells, promoting the release of tumor antigens and the activation of immune-mediated death of tumor cells, which in turn stimulates a peripheral systemic immune response that is potentially amplified by the administration of ICIs. In contrast, the survival benefit for patients’ candidates for second-line treatment options (regorafenib/cabozantinib or ramucirumab), although significant, is still modest. Thus, nivolumab with or without ipilimumab and pembrolizumab received FDA approval as second-line treatments. 

In addition to locoregional and systemic treatments, liver transplantation (LT) remains the better treatment option for a subset of patients with HCC, since the surgical procedure removes both the tumor and the liver at the same time, which remains the potential source of new neoplastic clones. The Milan criteria (MC) were developed more than 25 years ago and are still considered the benchmark for LT in patients with HCC. However, the strict application of MC might exclude some patients who may receive a clinical benefit from LT. Several expanded criteria have been proposed. Some consider pretransplant morphological and biological variables of the tumor, others consider post-LT variables such as the histology of the tumor, and others combine pre- and post-LT variables. More recently, the HCC response to locoregional treatments before transplantation emerged as a surrogate marker of the biological aggressiveness of the tumor to be used as a better selection criterion for LT in patients beyond the MC at presentation. These issues have been comprehensively updated in this *JCM* Special Issue [21] to present new policies that may be applied to better select patients with HCC for LT. The main innovative approach to select patients for LT presenting at baseline beyond the MC is to evaluate the characteristics and the duration of tumor response after locoregional (or systemic) therapies (downstaging treatment) and consider it a surrogate marker of biological HCC aggressiveness and of the risk of recurrence. It is mandatory to assess the success of downstaging treatments, to confirm the absence of tumor progression during an observation period of at least 3 months after treatment. Patients experiencing a successful downstaging are those eligible for LT as they present a less aggressive tumor biology and a better post-LT survival. Thus, the American and European associations for the study of the liver guidelines are concordant in recommending the adoption of locoregional (systemic) treatment procedures in patients with HCC beyond MC at baseline and the consideration of those who achieved successful downstaging for at least 3–6 months as suitable candidates for LT [22,23].

HCC represents approximately 50% of the indications for LT in Europe and the US. Constant indications for LT are decompensated liver cirrhosis due to cholestatic and autoimmune liver diseases, as well as chronic HBV infection. Decompensated cirrhosis due to chronic HCV infection is declining as an indication for LT, while alcohol- and non-alcoholic steato-hepatitis (NASH)-related liver diseases have increased progressively as indications for LT in recent years. In addition to the established indications for LT, clinical conditions historically considered exclusion criteria for LT, such as severe alcoholic hepatitis (AH), acute-on-chronic liver failure (ACLF), colorectal cancer metastases and cholangiocarcinoma, have been considered new indications for LT in recent years, producing promising survival advantages for patients. This topic has been highlighted in a very updated review [24] presented in this issue of *JCM*, where pros and cons for every new potential indication for LT have been critically discussed. Importantly, all newer indications for LT increase the pressure in an already difficult context of organ shortage. Strategies are therefore needed to increase the pool of transplantable organs, aiming to ensure a better balance between new candidate patients and available resources (organs). Moreover, a very challenging issue will be to optimize the patient selection criteria to ensure a clear gain in life expectancy for those who undergo LT, avoiding the increase in waiting list mortality for those patients who continue to await LT. A multidisciplinary transplant team is needed soon to face and solve this very delicate problem. Furthermore, the new scenario of transplants makes it essential to review and standardize ethical considerations across countries to ensure the same treatment options for all patients.

The COVID-19 pandemic has completely disrupted the global landscape of health systems. The repercussions have been highlighted in all sectors and in that of liver diseases. Data regarding the effect of COVID-19 on LT recipients are still scarce and often contradictory. A recent systematic review [25] presented in this issue of *JCM* showed that the COVID-19 clinical outcome of the LT population was not per se worse than that of the general population, although careful management of immunosuppressive therapy may be needed. In this regard, complete therapy discontinuation is not encouraged, but caution is needed in the use of mycophenolate mofetil (MMF), favoring tacrolimus (TAC) use. Anti-SARS-CoV-2 mRNA vaccine immunogenicity appeared to be low in LT patients, despite a booster dose being strongly recommended by the main scientific societies. The newest SARS-CoV-2 variants, such as Omicron, may further reduce vaccine-induced immunogenicity, suggesting that the level of surveillance should remain very high in this population.

A large body of new insights are derived from the collective work presented in this Special Issue of *JCM* entitled “New therapies for liver diseases”. All of them should be considered the beginning of a new era in exploring the pathophysiology of liver diseases and the mechanisms inducing cancer transformation of the liver with the help of technology, artificial intelligence and human perspectives. These new insights will promote the development of new and more effective treatments for several liver diseases that will improve quality of life and patient survival. As the Guest Editor of this Special Issue of *JCM*, I would like to express special thanks to the authors for their remarkable contributions and the reviewers for their professional comments. Furthermore, I would like to thank the *JCM* team for their professional and exceptional support that enabled the project to be achieved.
